# Supplementary material for: Integrative proteomic characterization of adenocarcinoma of esophagogastric junction
Source: Nat Commun. 2023 Feb 11;14:778. doi: 10.1038/s41467-023-36462-8 (PMC9922290; doi:10.1038/s41467-023-36462-8)
Supplement: Supplementary file 1 — Supplementary Information [file 41467_2023_36462_MOESM1_ESM.pdf]

Supplementary Information

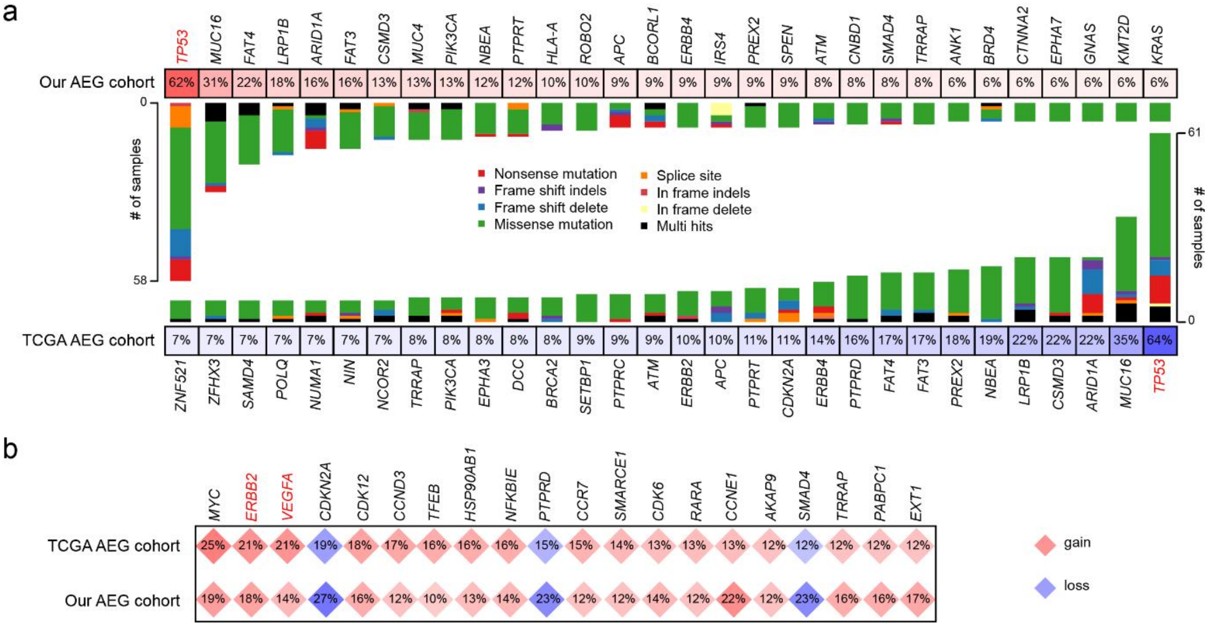

Supplementary Fig 1. Frequency genomic alterations in the TCGA and our AEG cohorts. a Top 30 mutated genes in the TCGA and our AEG cohorts. **b** Top 20 CNV genes in the TCGA AEG cohort and their frequency in our cohort.

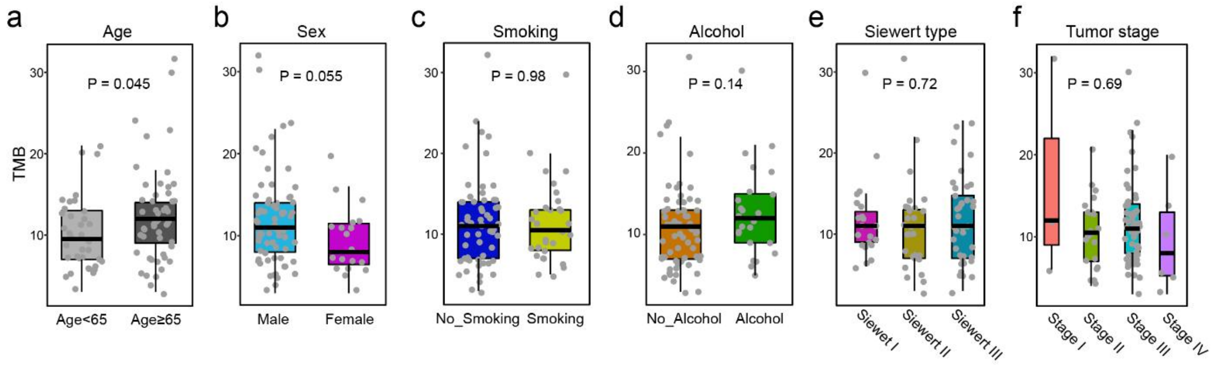

Supplementary Fig. 2 Box plots show the comparisons of TMB between patients with different clinicopathological features, including age (a), sex (b), smoking status (c), alcohol status (d), Siewert types (e), and tumor stages (f). P, Wilcoxon's rank-sum test. n = 36 for age < 65, n = 53 for age ≥ 65; n = 70 for male, n = 19 for female; n = 59 for non-smoking, n = 30 for smoking; n = 65 no alcohol, n = 24 alcohol; n = 22 for Siewert I, n = 29 for Siewert II, n = 38 for Siewert III; n = 3 for Stage I, n = 22 for Stage II, n = 58 for Stage III, n = 6 for Stage IV. Each box represents the IQR and median of TMB values in each group, whiskers indicate 1.5 times IQR.

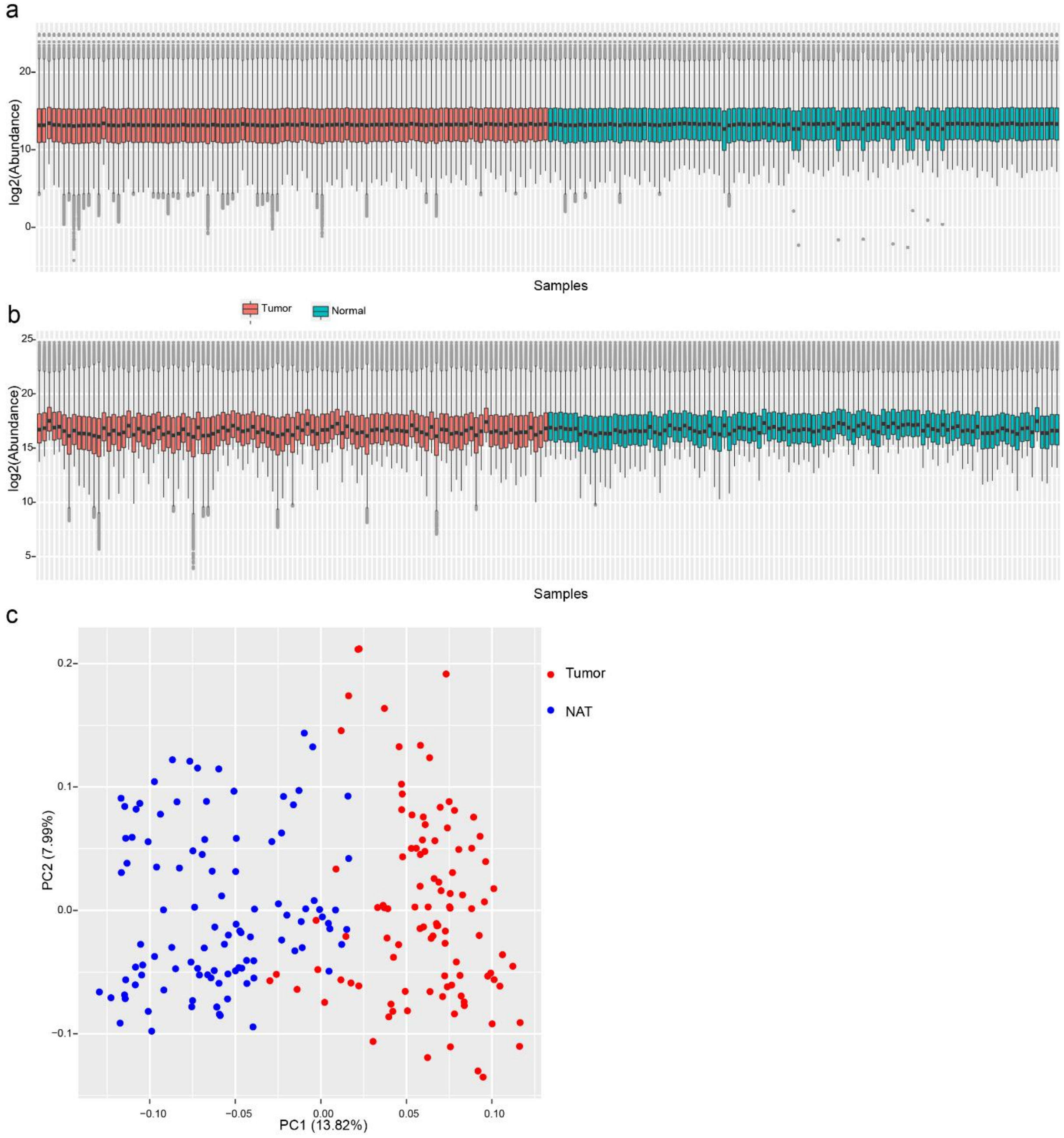

Supplementary Fig. 3 Quality assessment of proteomics and phosphoproteomics data. Distribution of relative abundance of detected proteins (**a**) and phosphorylation sites (**b**) across 206 samples. Salmon boxes indicate tumor samples and iris blue boxes represent NAT samples. **c** Principal component analysis (PCA) of protein abundances in paired AEG tumor and NAT samples. Red dots represent tumor samples and blue dots indicate NAT samples. In (**a**) and (**b**), each box represents the IQR and median of normalized abundance in each sample, whiskers indicate 1.5 times IQR.

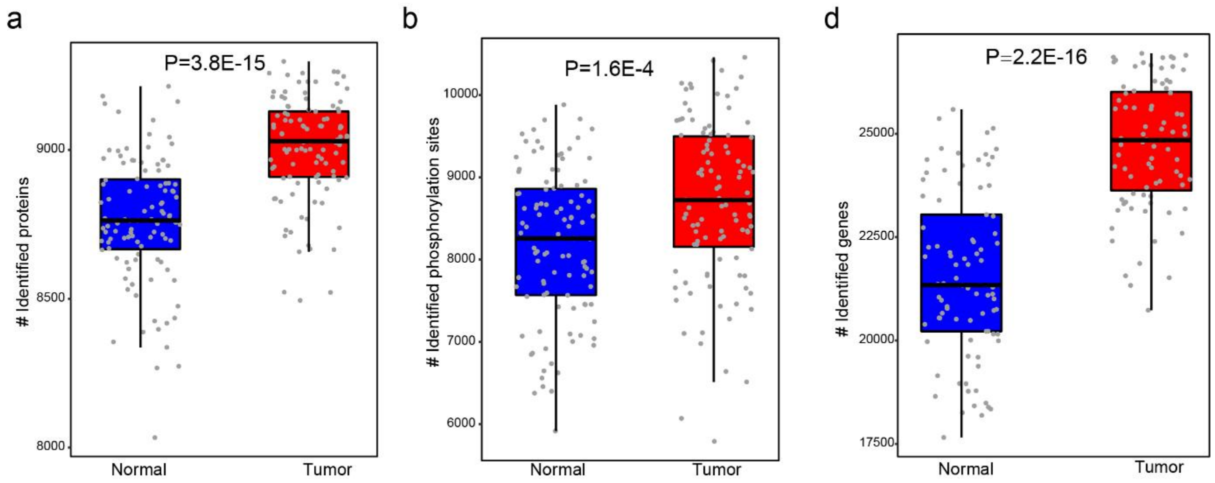

Supplementary Fig. 4 Comparisons of the numbers of identified proteins (a), phosphorylated sites (b), and genes (c) between paired AEG tumor and NAT samples. P, Wilcoxon's rank-sum test. Each box represents the IQR and median of the numbers of proteins, phosphorylation sites or genes in normal or tumor samples, whiskers indicate 1.5 times IQR. n = 103 paired tumor and normal samples.

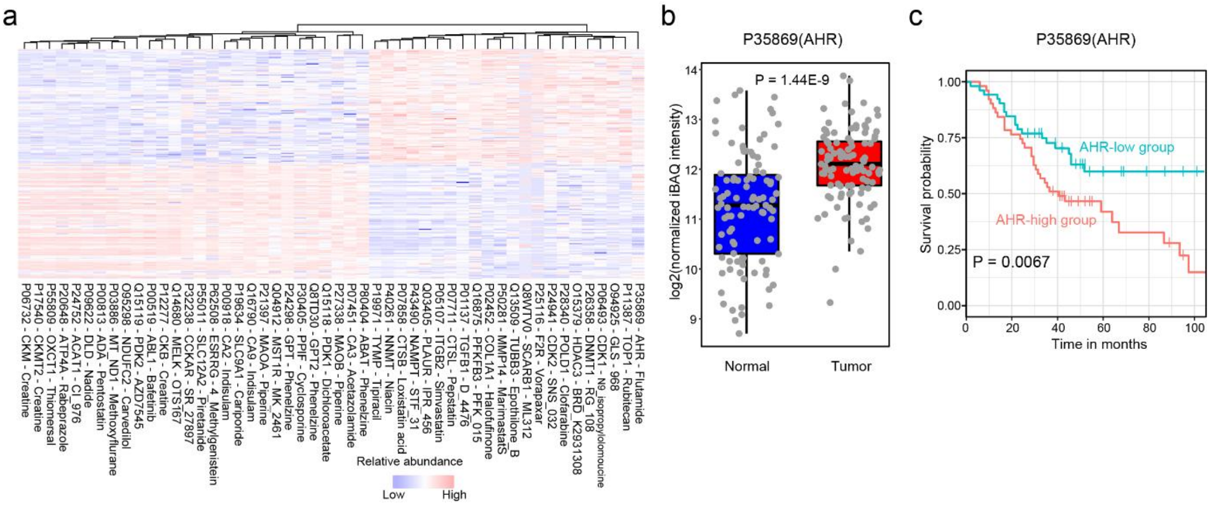

Supplementary Fig. 5 Top DEPs that are targeted by known drugs. a Heatmap showing the difference of the top 50 DEPs that are targeted by known anti-tumor compounds. **b** Box plot comparing the relative abundance of the AHR protein between AEG tumor and NAT samples (n = 103). P, Wilcoxon's rank-sum test. Each box represents the IQR and median of the normalized abundance in normal or tumor samples, whiskers indicate 1.5 times IQR. **c** Kaplan-Meier survival curve comparing overall survival time between groups that showed high- (n = 51) and low-abundance (n = 52) AHR protein. P, log-rank test.

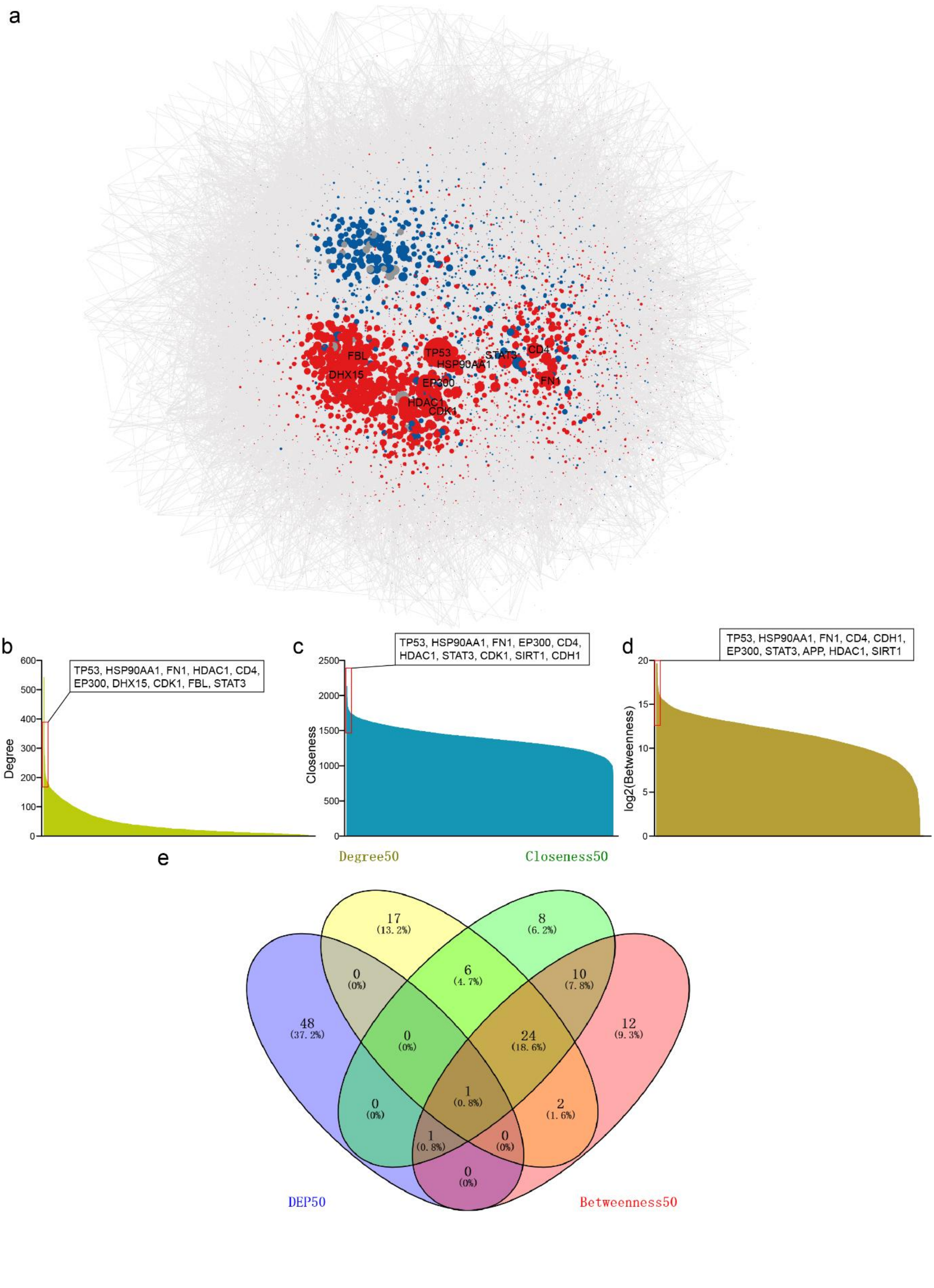

Supplementary Fig. 6 Network analysis of differentially expressed proteins (DEPs). a Protein-protein interactions (PPI) network of DEPs in AEG. The circle size indicates node degree. Red circle represents up-regulated protein, blue circle represents down-regulated

protein, and grey circle represents non-change protein. **b** The distribution of network degree of each node. Top 10 proteins with the largest degrees were highlighted. **c** The distribution of network closeness of each node. Top 10 proteins with the largest closeness were highlighted. **d** The distribution of network betweenness of each node. Top 10 proteins with the largest betweenness were highlighted. **e** Venny plot showing the overlaps between the top 50 DEPs and the top 50 proteins with the largest degree, closeness, or betweenness.

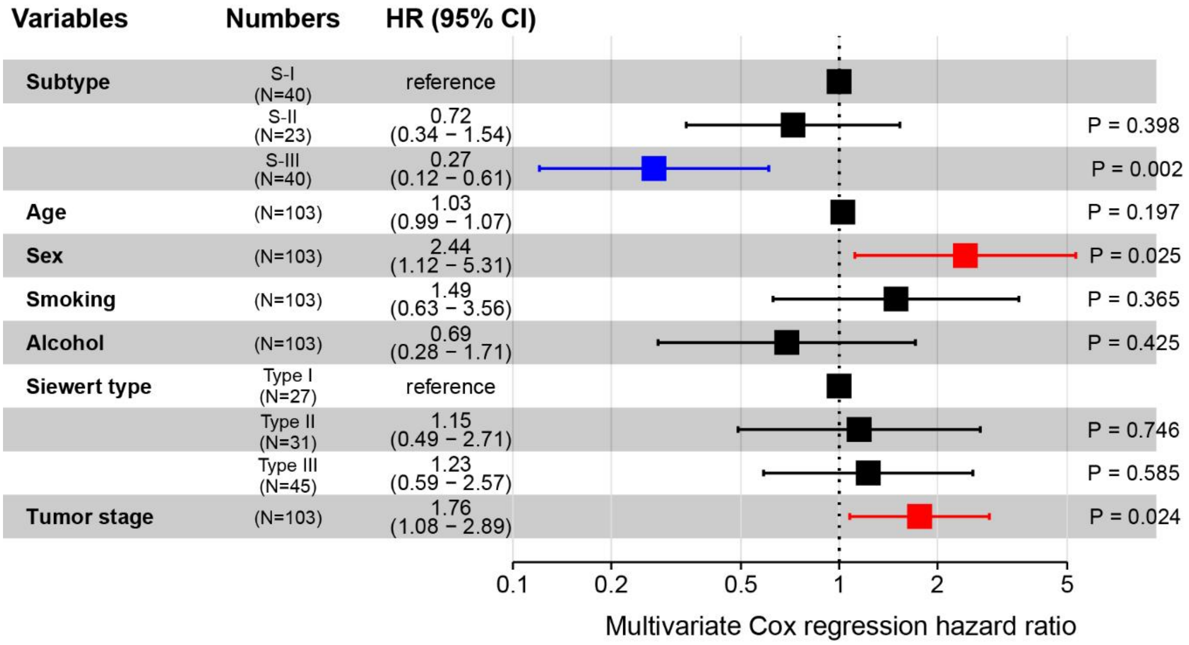

Supplementary Fig. 7 Multivariate Cox regression analysis of clinicopathological characteristics and the proteomics-based subtyping. P, multivariate Cox proportional hazard regression.

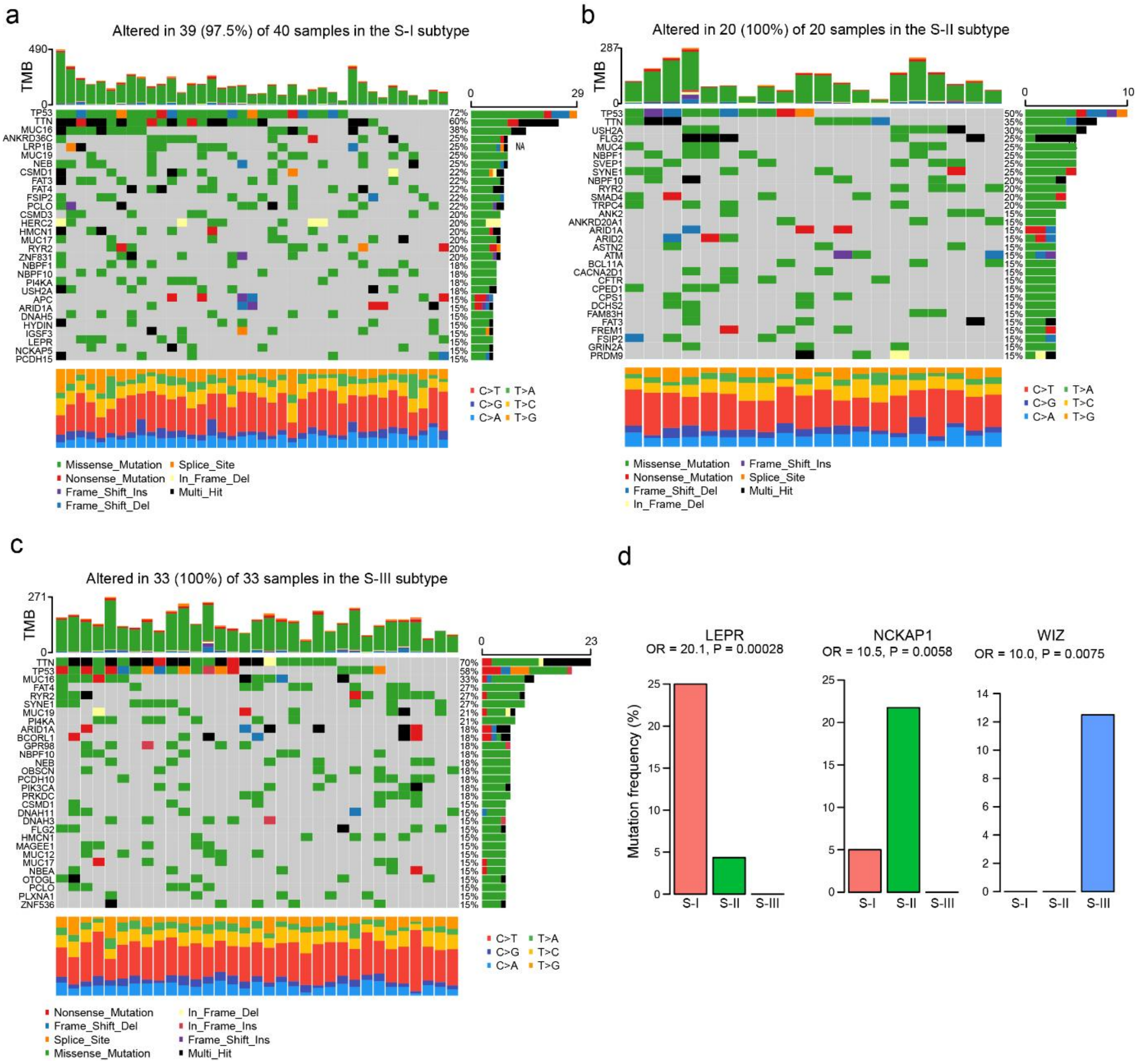

Supplementary Fig. 8 Comparisons of mutations among three proteomic AEG subtypes. Oncoplots show top 30 mutated genes in samples of the S-I subtype (**a**), S-II subtype (**b**), and S-III subtype (**c**). **d** Comparisons of mutation frequencies among three subtypes in the LEPR gene (specific in the S-I subtype), NCKAP1 gene (specific in the S-II subtype), and WIZ gene (specific in the S-III subtype). P, Fisher's exact test.

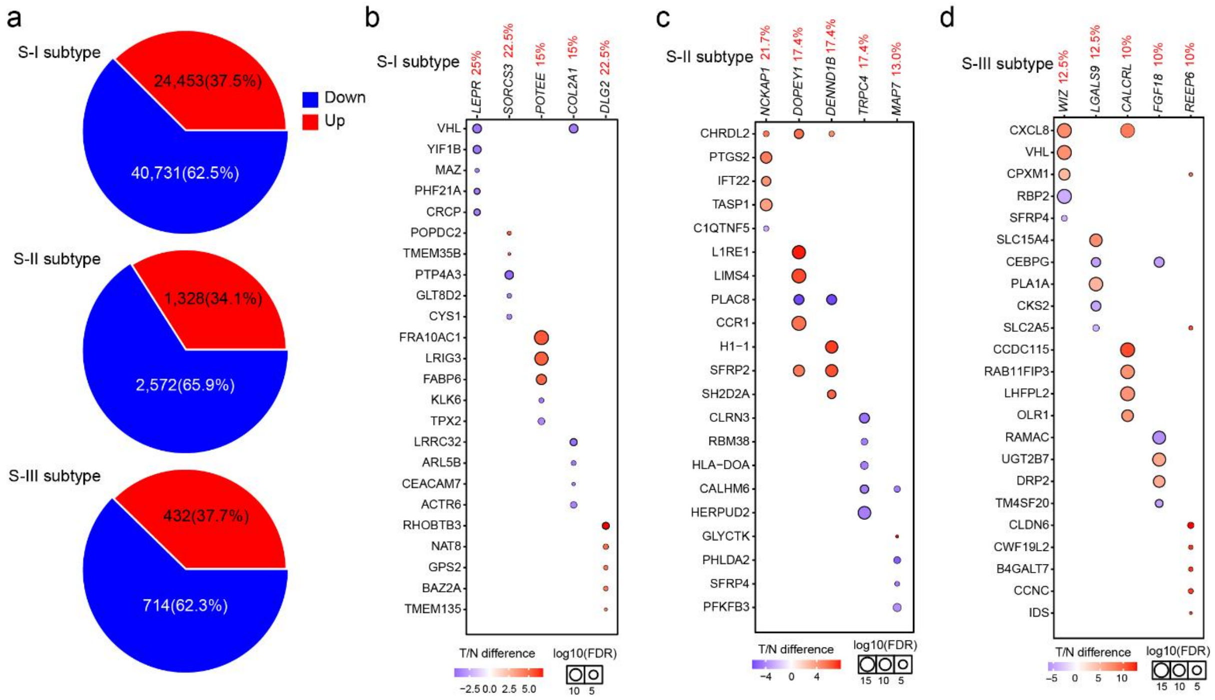

Supplementary Fig. 9 Significant effects of selected subtype-specific mutations on the proteins. a Pie charts show the percentages of up-regulated and down-regulated mutation-to-protein associations in the S-I subtype, S-II subtype, and S-III subtype, respectively. The top 5 mutation-protein associations of the top 5 mutated genes in the S-I subtype (**b**), S-II subtype (**c**), and S-III subtype (**d**).

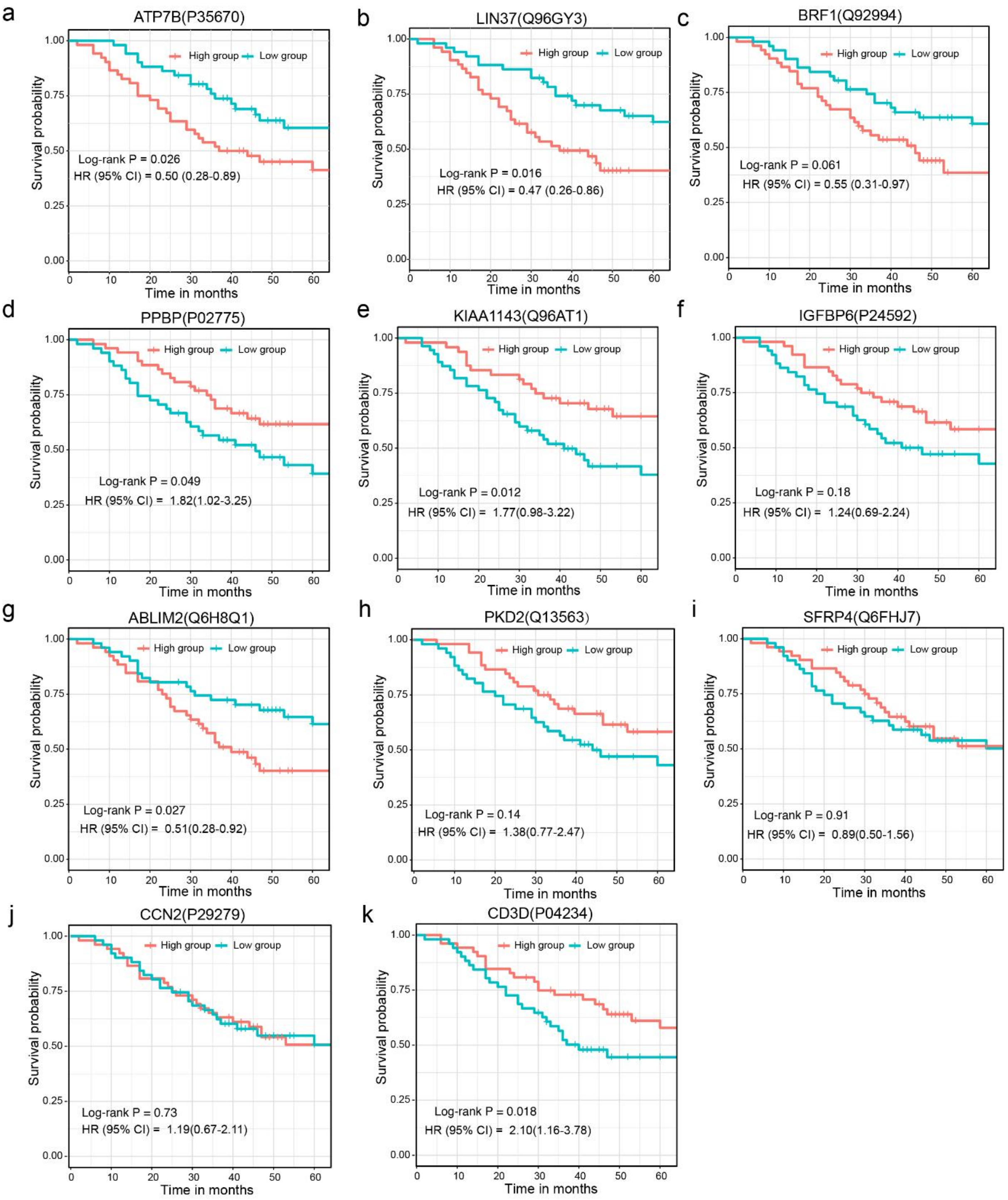

Supplementary Fig. 10 Kaplan-Meier survival curves of 11 signature proteins between corresponding high- and low-abundance patient groups, including ATP7B (a), LIN37 (b) , BRF1 (c), PPBP (d), KIAA1143 (e), IGFBP6 (f), ABLIM2 (g), PKD2 (h), SFRP4 (i), CCN2 (j), and CD3D (k). The result of FBXO44 is shown in Fig. 4c. n = 51 for high group and n = 52 for low group. P, log-rank test.

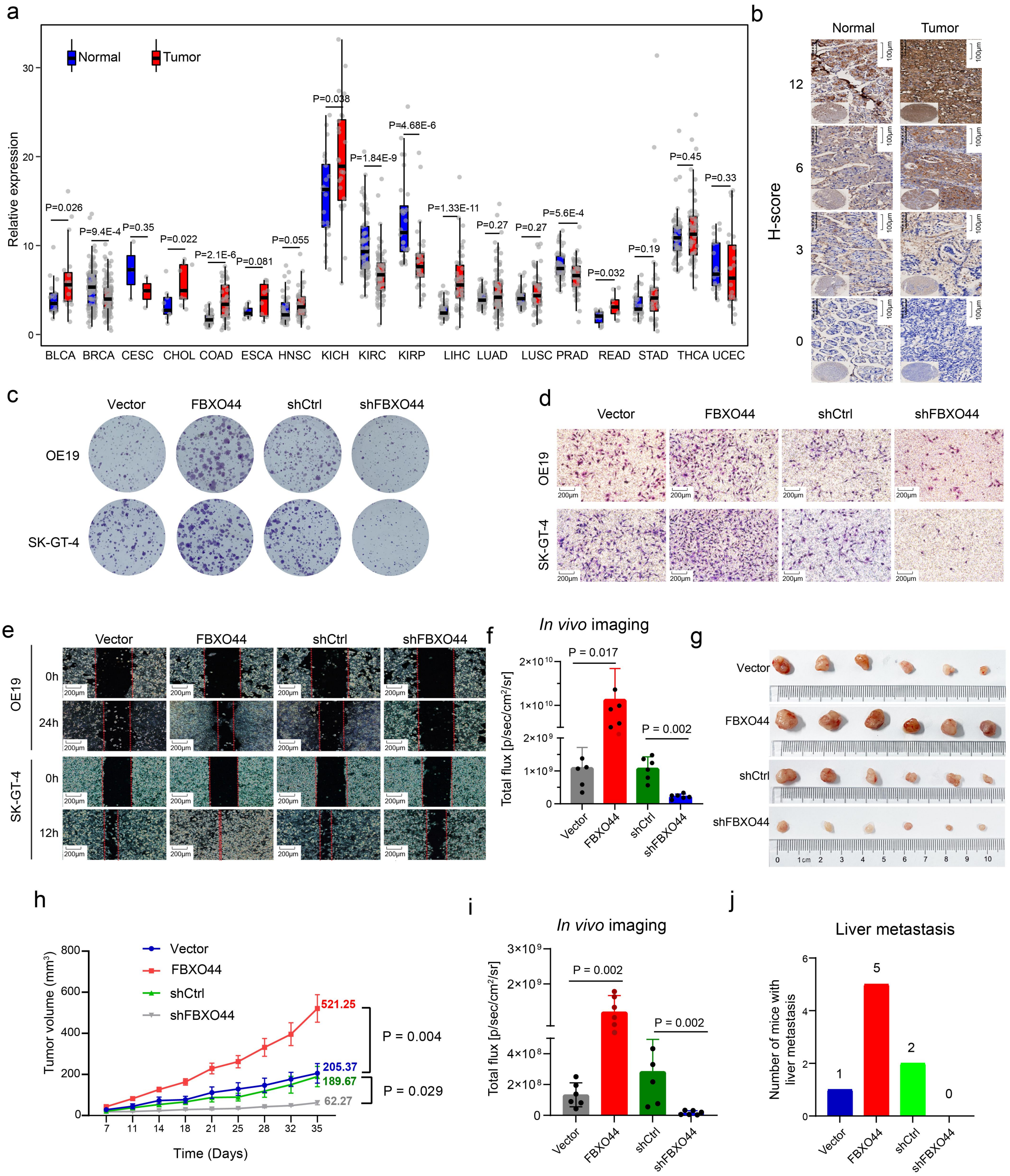

Supplementary Fig. 11 FBXO44 promotes AEG tumor progression and metastasis. a Comparisons of FBXO44 expression between paired tumor and NAT samples across 18 different tumor types in TCGA (n = 19 for BLCA, n = 112 for BRCA, n = 3 for CESC, n = 9 for CHOL, n = 41 for COAD, n = 10 for ESCA, n = 43 for HNSC, n = 24 for KICH, n = 72 for KIRC, n = 33 for KIRP, n = 50 for LIHC, n = 83 for LUAD, n = 56 for LUSC, n = 52 for PRAD, n = 9 for READ, n = 36 for STAD, n = 59 for THCA, n = 23 for UCEC). P, paired Student's t test. Each box represents the IQR and median of gene expression in normal or tumor samples of each tumor type, whiskers indicate 1.5 times IQR. **b** H-score system for evaluation of protein expression in tissues. Scale bar represents 100 μm. **c** Cell proliferation assays of FBXO44 overexpression (OE) and knockdown (KD) in the OE19 and SK-GT-4 cell

lines. **d** Transwell invasion assays of FBXO44 OE or KD in the OE19 and SK-GT-4 cell lines. Scale bar represents 200 μm. **e** Cell migration assays of FBXO44 OE or KD in the OE19 and SK-GT-4 cell lines. Scale bar represents 200 μm. **f** Quantitative analysis of *in vivo* imaging in Fig. 4h (n = 5 biological replicates). P, two-sided Student's t test. **g** Comparisons of tumor volumes among mice groups injected with OE19 cells transfected by vector, FBXO44 OE, KD control, and FBXO44 KD. **h** Quantitative analysis of tumor volumes in (**f**) (n = 5). P, two-sided Student's t test. **i** Quantitative analysis of *in vivo* imaging in Fig. 4i (n = 5 biological replicates). P, two-sided Student's t test. **j** The number of mice with liver metastasis in Fig. 4j. In (**f**, **h**, **i**), error bars represent mean ± SEMs. *P < 0.05, ** P < 0.01, and ***P < 0.001.

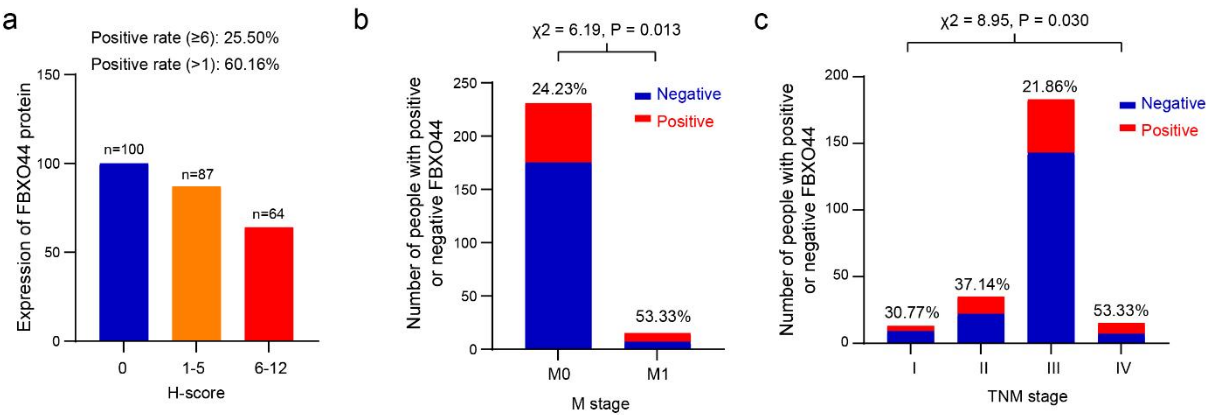

Supplementary Fig. 12 Validation of FBXO44 in an independent cohort of 251 AEG patients. a The number of samples with different H-score ranges (0, 1-5, and 6-12). **b** FBXO44 protein was significantly enriched in AEG patients with M1 tumor stage. P, Chi-square test. **c** FBXO44 protein was significantly enriched in AEG patients with advanced tumor stages. P, Chi-square test.

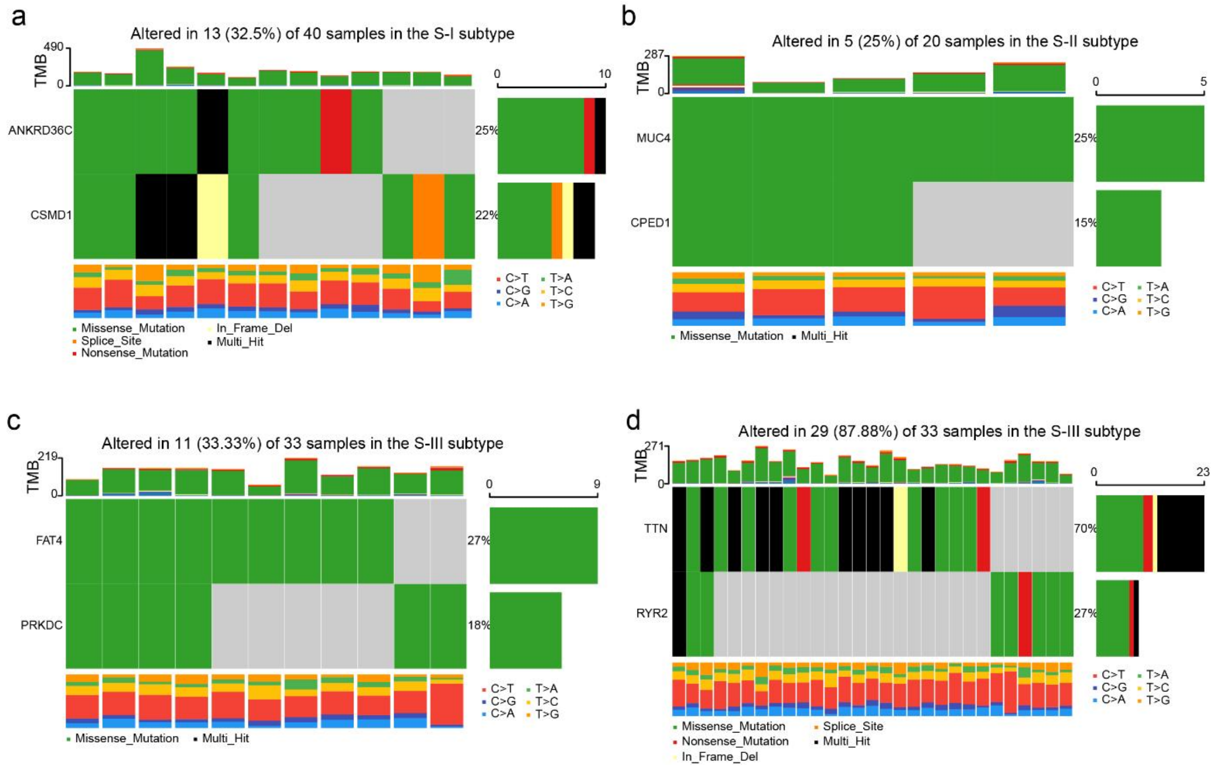

Supplementary Fig. 13 Examples of somatic interaction results. a The co-occurrence of ANKRD36C and CSMD1 mutations in the S-I subtype. **b** The co-occurrence of MUC4 and CPED1 mutations in the S-II subtype. **c** The co-occurrence of FAT4 and PRKDC mutations in the S-III subtype. **d** The mutually exclusive mutations between TTN and RYR2 in the S-III subtype.

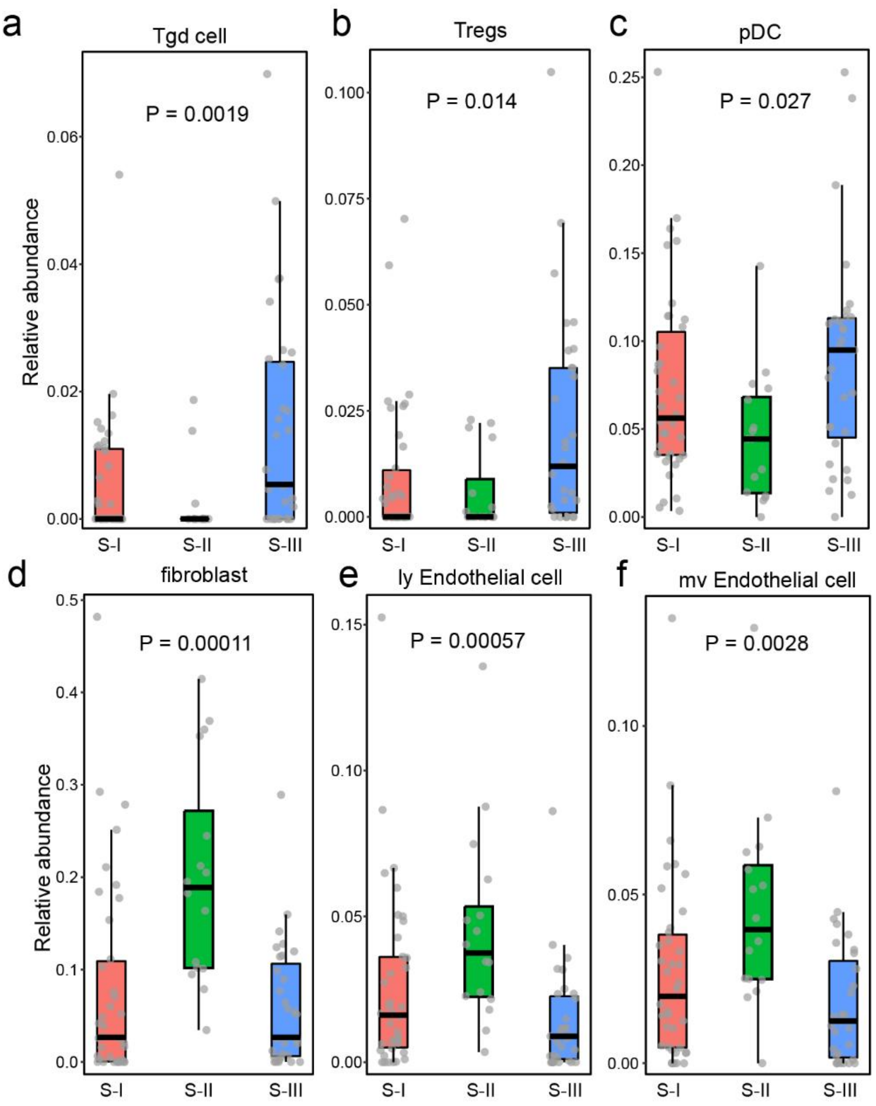

Supplementary Fig. 14. Box plots showing the differences in cell infiltration between the three AEG subtypes of Tgd cells (a), Tregs cells (b), pDC cells (c), fibroblasts (d), ly endothelial cells (e), and mv endothelial cells (f). P, Wilcoxon's rank-sum test. Each box represents the IQR and median of the relative cell abundance in each subtype, whiskers indicate 1.5 times IQR. n = 40 for S-I, n = 23 for S-II, n = 40 for S-III.

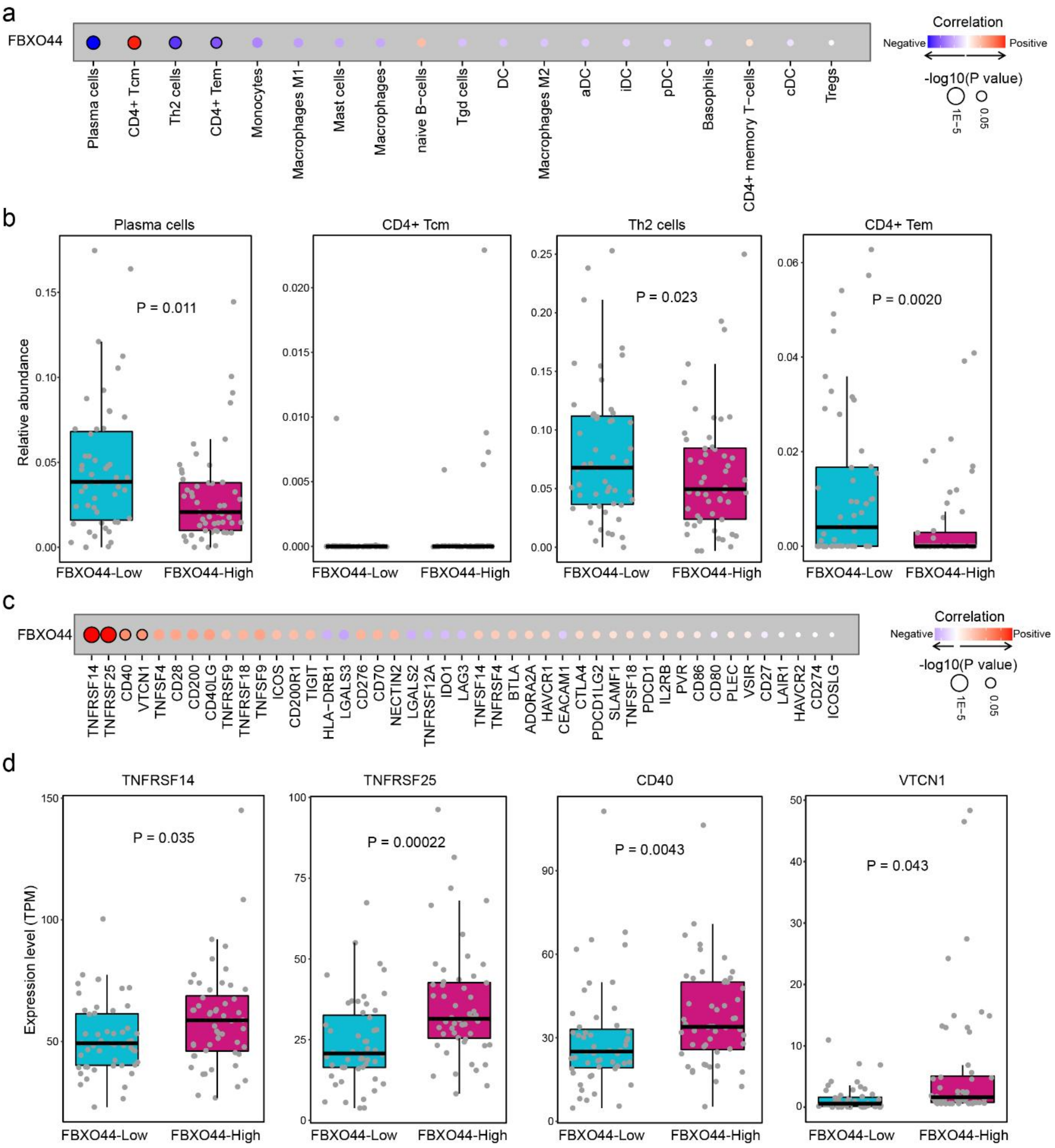

Supplementary Fig. 15 Associations of FBXO44 with immune cells and immune checkpoints. a The correlations between FBXO44 and different immune cells. **b** Box plots showing the relative abundance of plasma cells, $CD4^+$ Tcm, Th2 cells, and $CD4^+$ Tem between FBXO44-low and -high samples (n = 52 for FBXO44-high, and n = 51 for FBXO44-low). Each box represents the IQR and median of the relative cell abundances in FBXO44-low and FBXO44-high samples, whiskers indicate 1.5 times IQR. **c** The correlations between FBXO44 and immune checkpoint genes. **d** Box plots showing the expression level of TNFRSF14, TNFRSF25, CD40, and VTCN1 between FBXO44-low and -high samples (n = 52 for FBXO44-high, and n = 51 for FBXO44-low). P, Wilcoxon's rank-sum test. Each box represents the IQR and median of the gene expression in FBXO44-low and FBXO44-high samples, whiskers indicate 1.5 times IQR.

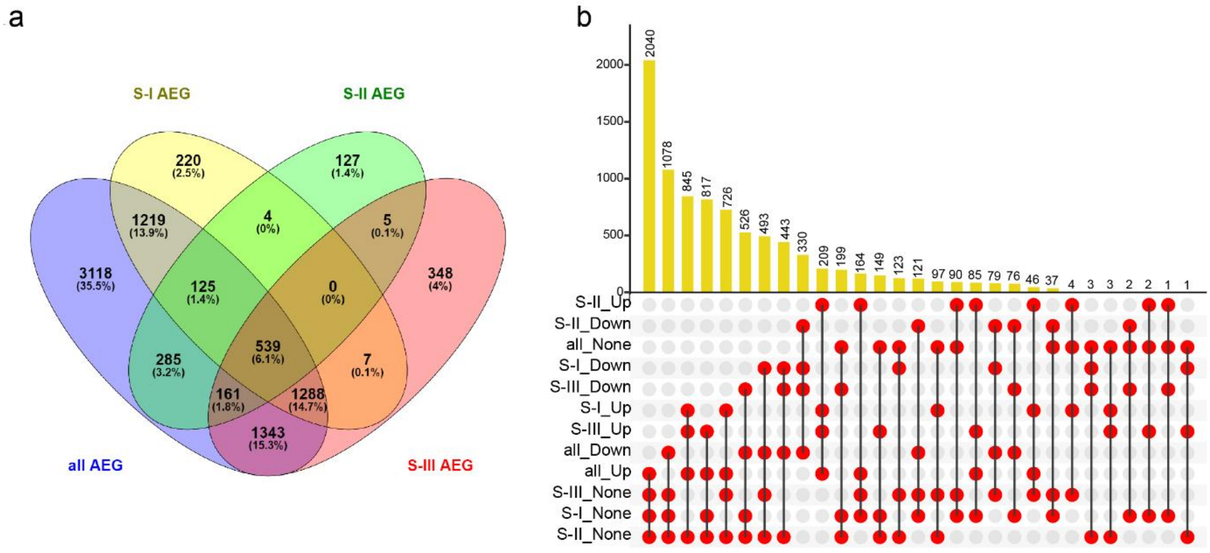

Supplementary Fig. 16 Differential phosphorylation sites in different comparisons. a Venny plot shows the overlaps among differential phosphosites identified in all AEG, S-I subtype, S-II subtype, and S-III subtype samples. **b** Upset plot shows the statistics of differential phosphosites in different comparisons.
