# Supplementary material for: Integrative proteomic characterization of adenocarcinoma of esophagogastric junction
Source: Nat Commun. 2023 Feb 11;14:778. doi: 10.1038/s41467-023-36462-8 (PMC9922290; doi:10.1038/s41467-023-36462-8)
Supplement: Supplementary file 3 — Description of Additional Supplementary Files [file 41467_2023_36462_MOESM3_ESM.pdf]

Description of Additional Supplementary Files

File Name: Supplementary Data 1
Description: Major clinical information of 103 AEG patients in this study.

File Name: Supplementary Data 2
Description: . SNVs across 94 AEG patients.

File Name: Supplementary Data 3
Description: Differentially expressed proteins in AEG

File Name: Supplementary Data 4
Description: Druggable Differential proteins.

File Name: Supplementary Data 5
Description: The degree, closeness, and betweenness of proteins in the differentially expressed protein PPI network.

File Name: Supplementary Data 6
Description: Subtyping information of 103 AEG tumor samples.

File Name: Supplementary Data 7
Description: Subtype-specific mutation genes.

File Name: Supplementary Data 8
Description: Significant mutation-to-protein associations in each subtype.

File Name: Supplementary Data 9
Description: The association between FBXO44 protein level and clinicopathological features of AEG patients.

File Name: Supplementary Data 10
Description: Differential phosphorylation sites in all AEG tumor and NAT samples.

File Name: Supplementary Data 11
Description: Differential phosphorylated sites in each AEG proteomic subtype.

File Name: Supplementary Data 12
Description: Correlations between known kinase-phosphosubstrate pairs.

File Name: Supplementary Data 13
Description: Protein group intensities across 103 AEG tumor samples and paired non-tumor samples.

File Name: Supplementary Data 14
Description: Gene counts across 85 AEG tumor samples and paired non-tumor samples.

File Name: Supplementary Data 15
Description: The scores and p values of different cell types generated from the xCell method.
