# Supplementary material for: Integrative proteomic characterization of adenocarcinoma of esophagogastric junction
Source: Nat Commun. 2023 Feb 11;14:778. doi: 10.1038/s41467-023-36462-8 (PMC9922290; doi:10.1038/s41467-023-36462-8)
Supplement: Supplementary file 19 — Reporting Summary [file 41467_2023_36462_MOESM19_ESM.pdf]

nature portfolio

Corresponding author(s): Xiang-Dong Cheng

Last updated by author(s): Nov 27, 2022

Reporting Summary

Nature Portfolio wishes to improve the reproducibility of the work that we publish. This form provides structure for consistency and transparency in reporting. For further information on Nature Portfolio policies, see our Editorial Policies and the Editorial Policy Checklist.

Statistics

For all statistical analyses, confirm that the following items are present in the figure legend, table legend, main text, or Methods section.

n/a	Confirmed	
☐	☒	The exact sample size (*n*) for each experimental group/condition, given as a discrete number and unit of measurement
☐	☒	A statement on whether measurements were taken from distinct samples or whether the same sample was measured repeatedly
☐	☒	The statistical test(s) used AND whether they are one- or two-sided<br>*Only common tests should be described solely by name; describe more complex techniques in the Methods section.*
☐	☒	A description of all covariates tested
☐	☒	A description of any assumptions or corrections, such as tests of normality and adjustment for multiple comparisons
☐	☒	A full description of the statistical parameters including central tendency (e.g. means) or other basic estimates (e.g. regression coefficient) AND variation (e.g. standard deviation) or associated estimates of uncertainty (e.g. confidence intervals)
☐	☒	For null hypothesis testing, the test statistic (e.g. *F*, *t*, *r*) with confidence intervals, effect sizes, degrees of freedom and *P* value noted<br>*Give P values as exact values whenever suitable.*
☒	☐	For Bayesian analysis, information on the choice of priors and Markov chain Monte Carlo settings
☒	☐	For hierarchical and complex designs, identification of the appropriate level for tests and full reporting of outcomes
☒	☐	Estimates of effect sizes (e.g. Cohen's *d*, Pearson's *r*), indicating how they were calculated

Our web collection on statistics for biologists contains articles on many of the points above.

Software and code

Policy information about availability of computer code

Data collection	No software was used for data collection.
Data analysis	MaxQuant (v 1.6.6.0), limmar R package (version 3.46.0), NMF R package (version 0.23.0), fastp (version 0.21.0), BWA (version 0.7.17),Picard tools (http://broadinstitute.github.io/picard/), Mutect2 (version 4.1.9.0), Strelka2 (version 2.9.10), Variant Effect Predictor (VEP) tool (release 102), maftools R package (version 2.6.5), KSEAapp (version 0.99.0), survival R package (version 3.2.3), survminer R package (version 0.4.9), Trimmomatic (version 0.39), HISAT2 (version 2.2.1), StringTie (version 2.14), GSVA R package (version 1.38.2), Cytoscape (version 3.9.0). Scripts and code that were used for data analysis and visualization were deposited in https://github.com/lishenglilab/AEG_Proteomics.

For manuscripts utilizing custom algorithms or software that are central to the research but not yet described in published literature, software must be made available to editors and reviewers. We strongly encourage code deposition in a community repository (e.g. GitHub). See the Nature Portfolio guidelines for submitting code & software for further information.

Data

Policy information about availability of data

All manuscripts must include a data availability statement. This statement should provide the following information, where applicable:

- Accession codes, unique identifiers, or web links for publicly available datasets
- A description of any restrictions on data availability
- For clinical datasets or third party data, please ensure that the statement adheres to our policy

The proteomics and phosphoproteomics data were deposited in the ProteomeXchange database85 with dataset identifiers PXD030667 (http://proteomecentral.proteomexchange.org/cgi/GetDataset?ID=PXD030667) and PXD030725 (http://proteomecentral.proteomexchange.org/cgi/GetDataset?ID=PXD030725), respectively. The WES and RNA-seq data were deposited in the Sequence Read Archive (SRA) database under the accession number PRJNA788008 (https://www.ncbi.nlm.nih.gov/sra/?term=PRJNA788008). The gene expression profiles, mutation, and CNV datasets of TCGA cohorts were retrieved from the Genomic Data Commons (GDC) data portal (https://portal.gdc.cancer.gov/). Software and publicly available resources used in this study were described in the Methods section. Other results generated in this study can be found in the supplementary data. Source data are provided with this paper.

Human research participants

Policy information about studies involving human research participants and Sex and Gender in Research.

Reporting on sex and gender	Only human biospecimens were used in this study. Sex is not considered in the study design.
Population characteristics	Patients in this cohort ranged from 40-87 years old; the cohort included 81 males and 22 females, 4 cases in stage I, 24 cases in stage II, 69 cases in stage III, and 6 cases in stage IV. We included 27 Siewert type I, 31 Siewert type II, and 45 Siewert type III AEG patients. More detailed clinical information of individual patients, including age, sex, smoking and drinking status, date of surgery, Lauren type, Borrmann classification, grade of differentiation, tumor size, tumor-node-metastasis (TNM) staging, and survival status and time, are listed in the Supplementary Data 1.
Recruitment	These patients were all newly diagnosed patients with AEG who underwent surgical resection and had received no prior treatment for this disease, including chemotherapy, radiotherapy, targeted therapy, or biological therapy. Patients who were found to have two or more malignancies were excluded.
Ethics oversight	The Research Ethics Committees of Zhejiang Cancer Hospital

Note that full information on the approval of the study protocol must also be provided in the manuscript.

Field-specific reporting

Please select the one below that is the best fit for your research. If you are not sure, read the appropriate sections before making your selection.

☒ Life sciences ☐ Behavioural & social sciences ☐ Ecological, evolutionary & environmental sciences

For a reference copy of the document with all sections, see nature.com/documents/nr-reporting-summary-flat.pdf

Life sciences study design

All studies must disclose on these points even when the disclosure is negative.

Sample size	We conducted proteomics and phosphoproteomics profiling in 103 AEG tumors with paired normal adjacent tissues(NATs), whole exome sequencing (WES) in 94 tumor-NAT pairs, and RNA sequencing (RNA-seq) in 83 tumor-NAT pairs. A total of 251 formalin-fixed, paraffin-embedded AEG tissues and corresponding NATs were collected for the immunohistochemical staining of FBXO44. These patients were all newly diagnosed patients with AEG who underwent surgical resection and had received no prior treatment for this disease, including chemotherapy, radiotherapy, targeted therapy, or biological therapy. Patients who were found to have two or more malignancies were excluded. Histological section obtained from the top and bottom portions of each specimen were reviewed by a senior board-certified pathologist to confirm the tissues as tumors or NATs. The top and bottom sections had to contain an average of 60% tumor cell nucleic with less than 20% necrosis to be deemed acceptable for this study. For cell line experiments, 5 biological replicates were used; For mice experiments, 6 biological replicates were used.
Data exclusions	No data exclusion.
Replication	All data analysis and experimental findings are reproducible. Three or six replicates were included, replicate numbers were provided in the corresponding legends.
Randomization	Randomization is not applicable to this study. This study is an observational study and does not need to adopt randomization.
Blinding	Blinding is not applicable to this study. This study is an observational study and does not need to adopt blinding.

Reporting for specific materials, systems and methods

We require information from authors about some types of materials, experimental systems and methods used in many studies. Here, indicate whether each material, system or method listed is relevant to your study. If you are not sure if a list item applies to your research, read the appropriate section before selecting a response.

Materials & experimental systems

n/a	Involved in the study	
☐	☒	Antibodies
☐	☒	Eukaryotic cell lines
☒	☐	Palaeontology and archaeology
☐	☒	Animals and other organisms
☒	☐	Clinical data
☒	☐	Dual use research of concern

Methods

n/a	Involved in the study	
☒	☐	ChIP-seq
☒	☐	Flow cytometry
☒	☐	MRI-based neuroimaging

Antibodies

Antibodies used	FBXO44 (Proteintech 10626-1-AP). For immunohistochemistry assays, after treating with 3% H2O2/methyl alcohol solution for 10 min at room temperature, 5% normal goat serum buffer was used to block the tissue at 37 ℃ for 30 min. Slides were then incubated with primary antibodies at 4 ℃ overnight. After washing, the slides were incubated with biotin labeled goat anti-rabbit IgG and HRP-conjugated streptavidin at 37 ℃ for 1 h. Immunoreaction was visualized by diaminobenzidine (DAB) (Cat#ZLI-9065, ZSGB-BIO Corp., Shanghai, China). After DAB staining, all tissues were counterstained with hematoxylin (Cat#ZLI-9609 ZSGB-BIO Corp., Shanghai, China) dehydrated and then blocked. The FBXO44 (1:300) antibody was purchased from Proteintech (Chicago, USA).
Validation	Immunohistochemical analysis of paraffin-embedded human liver cancer tissue slide using 10626-1-AP (FBXO44 antibody) at dilution of 1:200 (under 10x lens). Heat mediated antigen retrieval with Tris-EDTA buffer (pH 9.0).

Eukaryotic cell lines

Policy information about cell lines and Sex and Gender in Research

Cell line source(s)	Human AEG cell lines, OE19 and SK-GT-4, were purchased from the Cobioer Biosciences Co. ltd (Nanjing, China).
Authentication	All cell lines were authenticated by STR profiling.
Mycoplasma contamination	All cell lines were confirmed negative for mycoplasma contamination.
Commonly misidentified lines (See ICLAC register)	No cell lines used in this study are commonly misidentified lines in the ICLAC register.

Animals and other research organisms

Policy information about studies involving animals; ARRIVE guidelines recommended for reporting animal research, and Sex and Gender in Research

Laboratory animals	Mouse, NOD-SCID, male, 4 weeks old. Mice were fed in the Specific Pathogen Free (SPF) barrier center at the animal experimental center of Zhejiang Chinese Medical University, under standard conditions of temperature (25 ± 2 ℃) and humility (50 ± 5%) in a 12 h light /12 h dark cycle with normal drink and food.
Wild animals	The study did not involve wild animals.
Reporting on sex	This study included 81 males and 22 females, which is in line with the epidemiology of AEG. The gender was determined by self-reporting.
Field-collected samples	The study did not involve samples collected from the field.
Ethics oversight	Animal experiments were approved by the Institutional Animal Care and Use Committee of Zhejiang Chinese Medical University.

Note that full information on the approval of the study protocol must also be provided in the manuscript.
